# Characterization of *Shigella sonnei* in Malaysia, an increasingly prevalent etiologic agent of local shigellosis cases

**DOI:** 10.1186/1471-2334-12-122

**Published:** 2012-05-20

**Authors:** Xiu Pei Koh, Chien Shun Chiou, Noni Ajam, Haruo Watanabe, Norazah Ahmad, Kwai Lin Thong

**Affiliations:** 1 Institute of Biological Science, Faculty of Science, University of Malaya, Kuala Lumpur, 50603, Malaysia; 2 Laboratory of Biomedical Science and Molecular Microbiology, Institute of Graduate Studies, University of Malaya, Kuala Lumpur, 50603, Malaysia; 3 Central Region Laboratory, Center for Research and Diagnostics, Centers for Disease Control, Taichung City, 40855, Taiwan; 4 National Institute of Infectious Diseases, Tokyo, Japan; 5 Bacteriology Unit, Institute for Medical Research, Kuala Lumpur, Malaysia

**Keywords:** *Shigella sonnei*, Biotype, Diversity, MLVA, PFGE, Resistotype

## Abstract

**Background:**

Shigellosis is a major public health concern worldwide, especially in developing countries. It is an acute intestinal infection caused by bacteria of the genus *Shigella*, with a minimum infective dose as low as 10–100 bacterial cells. Increasing prevalence of *Shigella sonnei* as the etiologic agent of shigellosis in Malaysia has been reported. As there is limited information on the genetic background of *S. sonnei* in Malaysia, this study aimed to characterize Malaysian *S. sonnei* and to evaluate the prospect of using multilocus variable-number tandem-repeat (VNTR) analysis (MLVA) for subtyping of local *S. sonnei.*

**Methods:**

Forty non-repeat clinical strains of *S. sonnei* isolated during the years 1997–2000, and 2007–2009 were studied. The strains were isolated from stools of patients in different hospitals from different regions in Malaysia. These epidemiologically unrelated strains were characterized using biotyping, antimicrobial susceptibility testing, pulsed-field gel electrophoresis (PFGE) and MLVA.

**Results:**

The two biotypes identified in this study were biotype a (n = 29, 73%) and biotype g (n = 11, 27%). All the 40 strains were sensitive to kanamycin, ceftriaxone and ciprofloxacin. Highest resistance rate was observed for streptomycin (67.5%), followed by tetracycline (40%) and trimethoprim-sulfamethoxazole (37.5%). All the *S. sonnei* biotype g strains had a core resistance type of streptomycin - trimethoprim-sulfamethoxazole - tetracycline whereas the 29 biotype a strains were subtyped into eight resistotypes. All the strains were equally distinguishable by PFGE and MLVA. Overall, PFGE analysis indicated that *S. sonnei* biotype a strains were genetically more diverse than biotype g strains. Cluster analysis by MLVA was better in grouping the strains according to biotypes, was reflective of the epidemiological information and was equally discriminative as PFGE.

**Conclusions:**

The *S. sonnei* strains circulating in Malaysia throughout the period studied were derived from different clones given their heterogeneous nature. MLVA based on seven selected VNTR loci was rapid, reproducible and highly discriminative and therefore may complement PFGE for routine subtyping of *S. sonnei*.

## Background

Shigellosis is an acute intestinal infection caused by bacteria of the genus *Shigella*. The main symptom of this infection is bloody diarrhoea and the minimum infective dose is as low as 10–100 bacterial cells due to relative resistance to stomach acid [1]. Shigellosis is a major public health concern worldwide, especially in developing countries [2]. The infection is most frequent in children, the elderly and the immunocompromised [2,3]. A more recent annual estimate of the shigellosis burden was at 90 million incidences and 108,000 deaths [4].

*Shigella* can be differentiated into four species or serogroups, *S. dysenteriaeS. flexneriS. boydii*, and *S. sonnei* based on biochemical properties and group-specific O antigens in the outer membrane of the cell wall. There is a shift in *Shigella* dominance from *S. flexneri* to *S. sonnei* in developing and developed countries [4-6]. In Malaysia, Lee and Puthucheary [7] and Banga Singh *et al*. [8] reported an increasing dominance of *S. sonnei* as the etiologic agent of shigellosis. Apart from the apparent dominance shift, several studies have described the spread of a well-defined pandemic *S. sonnei* clone since 1990s which is characterized by common features such as biotype *g*, a particular PFGE *Xba*I pulsotype, resistance pattern of streptomycin - trimethoprim-sulfamethoxazole - tetracycline, and the presence of a 2.2 kbp class 2 integron [9,10].

Bacterial subtyping is frequently applied for outbreak investigations and surveillance of infectious diseases. Strain-specific fingerprints generated are used to facilitate the identification of disease transmission routes and sources [11]. Biotyping is one of the earlier phenotypic subtyping methods applied to *S. sonnei*. The bacteria can be subdivided into five biotypes (a, d, e, f, and g) on the basis of biochemical properties. This method however is not sufficiently discriminative.

Several genotyping methods with higher discriminatory power such as pulsed-field gel electrophoresis (PFGE) have been applied to subtype *S. sonnei*[12-14]. Since PFGE is a gel-based method, it requires strict adherence to standardized protocol for reproducible results. Standardized PulseNet PFGE protocol has been useful for inter-laboratory comparison of results [15]. However, due to the monomorphic nature of *S. sonnei*, PFGE occasionally may not be able to distinguish epidemiologically unrelated *S. sonnei* isolates and is not appropriate for phylogenetic analysis of strains that have evolved over a longer time span [16,17].

Multilocus variable-number tandem-repeat (VNTR) analysis (MLVA) is a highly discriminative sequence-based subtyping tool and has been used to study the genetic relatedness among *S. sonnei*. This method is based on the inherent variability of short sequences that are organized as tandem repeats at multiple VNTR loci. The VNTR loci in *S. sonnei* were found to have different degrees of variability [17]. MLVA with four to eight highly variable VNTR loci exhibited a discriminatory power parallel to or higher than PFGE [18]. Furthermore, MLVA based on the combination of VNTR loci with different variability has also been successfully used for phylogenetic analysis of *S. sonnei* that have evolved over different timescales [17].

Although *S. sonnei* is becoming an important etiologic agent of shigellosis in Malaysia, there is limited information on the genetic background of local strains. Therefore, the objective of the study was to characterize local *S. sonnei* strains by analyzing their biotypes, antimicrobial resistance patterns and genotypes. In addition, the prospect of using MLVA for routine subtyping of local *S. sonnei* in comparison with PFGE was also evaluated.

## Methods

### Bacterial strains

A total of 70 *Shigella* strains isolated from 1997–2009 were obtained from the Institute for Medical Research, Malaysia. However, only 40 strains *S. sonnei* from epidemiologically unrelated shigellosis cases were recovered from years 1997–2000 and 2007–2009 and analyzed. The strains were isolated from stools of patients in different hospitals in Malaysia. Each strain was exclusively from one patient. The shigellosis cases were from different regions, although mainly from northern region of Peninsular Malaysia.

### Biotyping

Biotyping was performed using fermentation of rhamnose and xylose and hydrolysis of ortho-nitrophenyl-/I-D-galactopyranoside (ONPG), and biotypes were designated according to methods described by Nastasi *et al.*[19]. Five biotypes are defined (biotypes a, d, e, f, and g) based on the combination of the positive/negative biochemical reactions.

### Antimicrobial susceptibility testing

Antimicrobial susceptibility testing was performed using Kirby-Bauer disc diffusion method according to guidelines of the Clinical and Laboratory Standards Institute [20]. *E. coli* ATCC 25922 was used as the quality-control strain. The antimicrobials commonly used for treatment of shigellosis were tested using antimicrobial-impregnated discs from Oxoid, Basingstoke, UK: ampicillin (A, 10 μg), chloramphenicol (C, 30 μg), streptomycin (S, 10 μg), tetracycline (T, 30 μg), ciprofloxacin (Cip, 5 μg), kanamycin (K, 30 μg), ceftriaxone (Cro, 30 μg), nalidixic acid (N, 30 μg) and trimethoprim–sulfamethoxazole (Sx, 25 μg).

### Pulsed-field gel electrophoresis (PFGE)

All *S. sonnei* strains were analyzed with the restriction enzyme *Xba*I (Promega, Madison, WI, USA) using standardized PulseNet PFGE protocol, with extended run time of 25 hours in a CHEF Mapper system (Bio-Rad, U.S.A.) [15]. *Xba*I-digested *Salmonella* serotype Braenderup H9812 was used as the molecular weight standard. Computer-assisted analysis of the PFGE banding patterns was performed with BioNumerics software version 6.0 (Applied Maths, Kortrijk, Belgium). Banding patterns were compared based on the Dice coefficient, and a dendrogram based on the PFGE-*Xba*I profiles was constructed by unweighted pair group method with arithmetic averages (UPGMA) algorithm. A band position tolerance of 1.0% and optimization of 1.5% was applied during the comparison of banding patterns.

### Multilocus variable-number tandem-repeat analysis (MLVA)

Seven VNTR loci, SS1, SS3, SS6, SS9, SS10, SS11, and SS13 previously reported by Liang *et al.*[18] were used. The primers and conditions are indicated in Table 1. Two multiplex polymerase chain reactions (mPCR) were carried out; mPCR1 consisted of primers for SS1, SS3, SS6, and SS9 while mPCR2 consisted of primers for SS10, SS11, and SS13. Each 10 μl PCR mixture contained 1X PCR buffer, 3 mM MgCl_2_, 0.2 mM of each dNTPs, 0.05 to 0.2 μM of each primer, 1 unit of GoTaq® Flexi DNA polymerase (Promega, Madison, WI, USA), and 1 μl of DNA template. Crude DNA template was prepared as follows: A pure culture of *S. sonnei* was plated on Luria-Bertani agar and incubated overnight at 37°C. A single colony was removed from the plate, suspended in 100 μl of sterile deionized water, boiled for 5 min and immediately cooled on ice. After centrifugation at 12,100 *g* for 3 min, the supernatant was transferred into a new tube for subsequent PCR analysis. PCR conditions were as follows: initial denaturation at 94°C for 5 min; 30 cycles of denaturation at 94°C for 45 s, annealing at 55°C for 50 s and extension at 72°C for 60 s; and final extension at 72°C for 5 min.

**Table 1:** MLVA primers, number of alleles and Nei’s index of diversity (D)

Locus*	**Dye_Forward primer (5′ to 3′)**	**No. of alleles**	**Nei’s diversity index, D**
**SS1**	VIC_TTGCCAGTACACCTCACTCG	13	0.82
**SS3**	6-FAM_CTGGGAGATGAACAGGAGGA	16	0.92
**SS6**	NED_GAGTCGCTAAACGCTTGCTT	17	0.90
**SS9**	PET_CGCAATCAGCAAAACAAAGA	12	0.88
**SS10**	6-FAM_ACGGTGGGCTTTCTCTACCT	6	0.75
**SS11**	VIC_CTGGTCCGGGAGATTATCG	8	0.83
**SS13**	PET_AGACGCTGGCTTATGACGAT	3	0.42

* Liang *et al.* (2007).

The resulting PCR products were diluted 20 times with sterile deionized water and separated on an ABI 3130XL genetic analyzer using the size standard Gene Scan LIZ 500 (Applied Biosystems). Amplicon sizes were converted to copy numbers using BioNumerics software version 6.0 (Applied Maths, Kortrijk, Belgium). Each unique allelic string was designated a unique MLVA type. A dendrogram was constructed by UPGMA clustering based on categorical coefficient. Minimum spanning tree algorithm was used to construct a minimum spanning tree (MST) to determine phylogenetic pattern. The allelic profiles of 173 strains from Taiwan, China, Indonesia, Vietnam, and Cambodia, previously reported by Chiou *et al.*[16] and Liang *et al.*[18] were extracted and compared to the present data in the MST analysis based on the seven tested loci.

The polymorphism of each VNTR locus was represented by Nei’s diversity index, calculated as 1 - ∑ (allelic frequency)^2^. Simpson’s index of diversity (*D*) and Non-Approximated Confidence Interval (C.I.N.A.) were calculated as described previously by Simpson [21] to compare the discriminatory powers of PFGE and MLVA.

## Results

### Biotypes of *S. sonnei*

Two biotypes were identified among the *S. sonnei* strains, i.e. biotype *a* (ONPG +, rhamnose +, xylose –) and biotype *g* (ONPG +, rhamnose –, xylose –). Out of the 40 strains tested, 29 (73%) and 11 (27%) were of biotypes *a* and *g*, respectively.

### Antimicrobial susceptibility profiles

**Figure 1:**
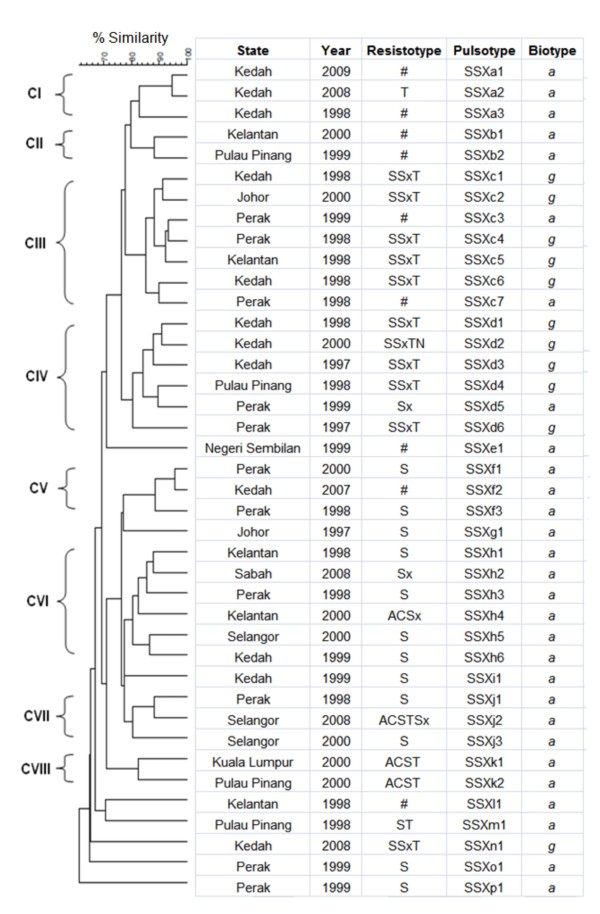
**Dendrogram of*S. sonnei*PFGE patterns generated by UPGMA clustering method using Dice coefficient.** Key: all sensitive (#), ampicillin (A), chloramphenicol (C), streptomycin (S), tetracycline (T), and trimethoprim–sulfamethoxazole (Sx).

All the 40 strains were sensitive to kanamycin, ceftriaxone and ciprofloxacin. The resistance rates of other antimicrobials are as follows: streptomycin (67.5%), tetracycline, (40%), trimethoprim-sulfamethoxazole (37.5%), ampicillin (10%), chloramphenicol (10%) and nalidixic acid (2.5%). Nine strains (22.5%) were susceptible to all the antimicrobials tested. Fifteen strains (37.5%) were multidrug-resistant (MDR, defined as resistance to three or more classes of antimicrobials) with resistotype SSxT being dominant (n = 10), followed by ACST (n = 2), ACSx (n = 1), SSxTN (n = 1) and ACSTSx (n = 1). Resistotype SSxT was exclusively associated with 10 out of 11 *S. sonnei* biotype *g* strains. The remaining biotype *g* strain showed additional resistance towards nalidixic acid besides having a core resistance type of SSxT. On the contrary, the biotype *a* strains have eight different resistotypes (Figure 1).

### Genotypes based on PFGE

The genetic relatedness of the strains was determined by PFGE. *Xba*I-digested *S. sonnei* DNA generated 40 reproducible unique PFGE patterns, each with 15 – 24 DNA bands, with a Dice coefficient, F ranging from 0.70 to 0.98. Based on 80% similarity, eight clusters (CI – CVIII) were observed (Figure 1). Most clusters contained strains from different geographical regions. Similarly, most clusters contained strains isolated in different years except cluster CVIII which contained two biotype *a* strains isolated in year 2000 with identical resistotype ACST. Cluster CI contained three biotype *a* strains from Kedah. Cluster CII contained two biotype *a* strains which were sensitive to all the antimicrobials tested. Ten of the 11 biotype *g* strains were dispersed in cluster CIII and cluster CIV. Cluster III contained five biotype *g* and two biotype *a* strains. One of the biotype *a* strain even shared 93.3% similarity with a biotype *g* strain. Another five biotype *g* strains were grouped in cluster CIV with one biotype *a* strain. Cluster V contained three biotype *a* strains. Two strains in this cluster were closely related to each other (95.5% similarity) despite being isolated seven years apart from two different locations (Perak and Kedah). Cluster VI and cluster VII contained six and three biotype *a* strains, respectively. Seven biotype *a* and one biotype *g* strains were not assigned into any of these clusters.

### Genotyping by MLVA

**Figure 2:**
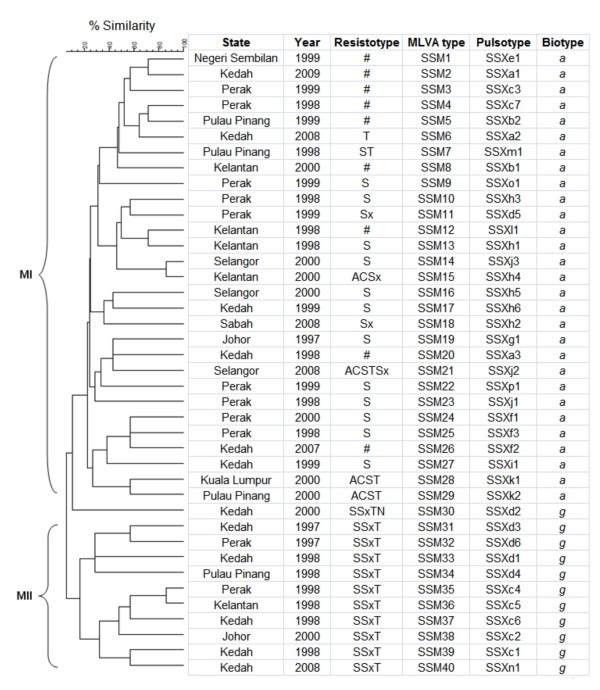
**Dendrogram generated from*S. sonnei*MLVA suptyping by the UPGMA clustering method using categorical coefficient.** Key: all sensitive (#), ampicillin (A), chloramphenicol (C), streptomycin (S), tetracycline (T), and trimethoprim–sulfamethoxazole (Sx).

**Figure 3:**
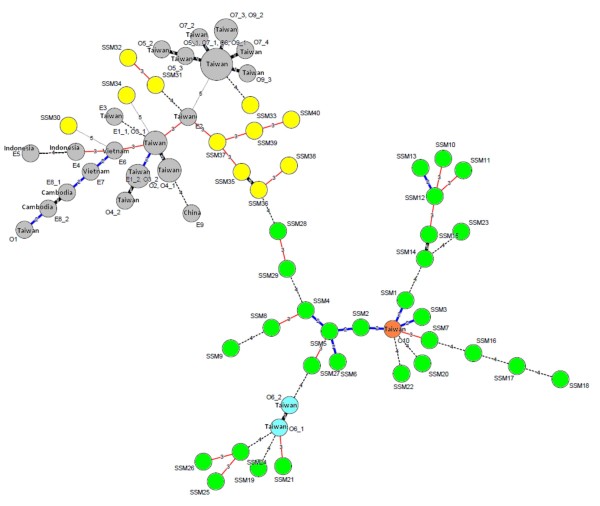
**Minimum spanning tree for the 40 Malaysian*Shigella sonnei*strains and strains from five other countries.** For Malaysian strains, each circle represents one strain with a unique MLVA profile. Each circle is noted with the MLVA type of the strain. MLVA types in yellow colour are Malaysian biotype *g* strains whereas those in green are Malaysian biotype *a* strains. For the profiles of strains from five other countries, the circle size is not proportional to the number of strains belonging to the MLVA profile but it is proportional to the number of outbreaks or unrelated episodes sharing the same profile. Circles in grey colour denote global clonal group A, orange denotes group B, and blue denotes group C. Additional information on these foreign strains is provided [see Additional file 1]. The distance between two MLVA types is numbered. A distance of one locus between two closest MLVA types is marked by a thick black line, a distance of two loci is marked by a blue line, and a distance of three loci is indicated by a thin red line. A distance of four loci is indicated by a broken line and a distance of five loci is indicated by a light broken line.

MLVA based on seven VNTR loci was performed to further characterize the *S. sonnei* strains. The number of alleles and diversity of each locus are listed in Table 1. Among the seven loci tested, SS1, SS3, SS6, SS9, and SS11 were more variable compared to SS10 and SS13. Locus SS13 showed the lowest diversity (D = 0.42) while SS3 was the most diverse (D = 0.92). From our result, the biotype *a* strains shared a single common allele at locus SS13 whereas two common alleles were detected at this locus for *S. sonnei* biotype *g*. The combination of the seven VNTR loci with high and low degree of variability was sufficient for molecular subtyping of the strains. The 40 *S. sonnei* strains were discriminated into 40 different MLVA types. Two pairs of single-locus variants, SSM14 and SSM15, and SSM35 and SSM36 were detected. The strains in each of the pair were isolated in the same year but from different regions. Two main clusters, MI and MII were observed from the dendrogram generated (Figure 2). Cluster MI consists of biotype *a* strains while cluster MII consists only biotype *g* strains. There was no clear demarcation of the strains isolated from different years and localities. From the MST, it is apparent that the *S. sonnei* in this study were heterogeneous as the tree branched out extensively. Most MLVA types have a distance of two to four loci from each other and the biotype *a* and *g* strains were differentiated into two main branches (Figure 3). The low variability locus SS13 established the ‘trunk’ of the MST, separating the biotype *a* and *g* strains while the six remaining highly variable loci further discriminated the strains in each branch, establishing the ‘twig and leaves’ of the tree.

*S. sonnei* is one of the major etiologic agents of travel-associated diarrhoea [22]. Therefore our results were compared with MLVA profiles of 173 strains from five other Asian countries to determine if these strains were closely related. Analyses of MST (Figure 3) show that most of the Malaysian biotype *g* strains were related to the Taiwanese strains than those strains isolated from neighbouring countries (Indonesia, Vietnam, and Cambodia). Only one Malaysian biotype *g* strain was distantly related to the Vietnamese strains, sharing common alleles at two VNTR loci. The biotype *a* strains were also related to strains from Taiwan. In particular, three Malaysian biotype *a* strains shared common alleles at five VNTR loci with the Taiwanese strains.

### Comparison of discriminatory power of MLVA and PFGE in genotyping of *S. sonnei*

PFGE and MLVA displayed excellent resolution in discriminating the Malaysian *S. sonnei*. Both methods were equally discriminative with a Simpson’s index of diversity, D of 1.0 (C.I.N.A. 0.989-1.011).

## Discussion

The predominant biotype identified in this study was biotype *a* (n = 29, 73%) and the remaining strains were of biotype *g* (n = 11, 27%). These two biotypes were the most commonly reported biotypes in Australia from 1990–2009 [23]. In many other countries, an increasing prevalence of *S. sonnei* biotype *g* was reported. Izumiya *et al.*[14] reported the prevalence of *S. sonnei* biotype *g* among travel associated cases in Japan while almost all biotype *a* isolates were from patients with travel history to Southeast Asia. In Korea, *S. sonnei* isolated during 1977–1986 were of biotype *a*, whereas isolates in 1991–2000 were of biotype *g*[24]. Similarly, *S. sonnei* isolated from cases acquired in Ireland, Italy, United States, and a few African countries also suggested the increasing prevalence of *S. sonnei* biotype *g* since the 1990s [9,25,26]. More Malaysian strains however need to be analyzed to obtain a more accurate representation of the distribution and prevalence of *S. sonnei* biotypes in Malaysia.

Overall, PFGE analysis in this study indicated that *S. sonnei* biotype *a* strains were genetically more diverse than biotype g strains. The non-rhamnose fermenting biotype *g* strains were given further attention as there is a spread of a pandemic clone of biotype *g* strains across different continents [9,10]. Ten out of 11 Malaysian biotype *g* strains in this study exhibited similar characteristics of the pandemic clone that is biotype *g,* resistance pattern of SSxT, and similar but distinguishable PFGE patterns. A number of these biotype *g* strains were highly similar in PFGE banding patterns. The banding patterns, by visual comparison, were similar to some biotype *g* strains from Ireland and Italy [13]. These Malaysian strains could be phylogenetically linked to the pandemic biotype *g* clone. The biotype *g* strains were isolated from the period 1997 to 2000 and year 2008, indicating that these strains persisted in Malaysia for at least a decade.

In this study, more than half of the strains were resistant towards streptomycin (67.5%) and all strains were susceptible to kanamycin. This is in agreement with an earlier study in Malaysia by Hoe *et al.*[12] on Malaysian strains collected during the period 1997–2000. Resistance to trimethoprim-sulfamethoxazole and tetracycline are commonly reported in *S. sonnei*[5,14,24,27]. However the resistance rates to trimethoprim-sulfamethoxazole and tetracycline in this study were relatively low at 37.5% and 40%, respectively as compared to those observed in Taiwan, Thailand, Japan and Korea [5,14,24,27]. Most of the strains in this study remained susceptible to ceftriaxone, ciprofloxacin, ampicillin, chloramphenicol and nalidixic acid. These results were consistent with a study on *S. sonnei* from Northeast Malaysia [8]. The low resistance rate to nalidixic acid in Malaysia is in concordance with a report by Izumiya *et al.*[14] where resistance to nalidixic acid was less frequent in *S. sonnei* originated from South-east Asia. MDR *S. sonnei* strains persisted throughout the study periods. Selective antibiotic pressure may lead to the persistence of MDR *S. sonnei* strains in Malaysia [12] as ampicillin and trimethoprim-sulfamethoxazole are used for the treatment of *Shigella* infection locally [28]. To the best of our knowledge, all the 40 infected patients recovered from their illness.

PFGE and MLVA showed comparable discrimination in subtyping of *S. sonnei.* Both techniques further differentiated the *S. sonnei* biotype *a* and *g* strains. However MLVA was better at grouping the strains on the basis of biotypes, and the overall percentage of similarity among strains was low when subtyping was done using MLVA. Furthermore, MLVA subtyping was more rapid and less laborious compared to PFGE. Interpretation of result was less subjective and results were more readily comparable between laboratories. All these suggest that MLVA may be a suitable complement to PFGE or even an alternative for routine subtyping of *S. sonnei*.

Most of the PFGE and MLVA clusters contained strains from multiple geographical locations, some clusters even contained strains isolated from distant parts of the country although they were epidemiologically unrelated*.* This concurred with the report of Pichel *et al.*[3] where epidemiologically unrelated Argentinean *S. sonnei* from very distant geographical areas were clustered together by PFGE. Epidemiologically unrelated MLVA single-locus variants, and strains isolated seven years apart at different locations yet with highly similar PFGE pattern were also observed. These observations where strains with no apparent epidemiological linkage were clustered together may be due to travel of individuals within Malaysia, long-persisted dissemination of different clones of *S. sonnei* throughout the country, person-to-person transmission of a particular strain over an extended period with minor genetic changes, or a combination of these events.

Based on the MST analysis, *S. sonnei* in the present study were heterogeneous, and a number of strains in this study were related to *S. sonnei* from Taiwan and to a lesser extent, to those from neighbouring countries. Since *S. sonnei* is frequently found responsible for travel-associated diarrhoea, it is not uncommon that transmission of *S. sonnei* across countries and even continents occurred easily. A study on the global distribution of *S. sonnei* clones divided the strains from 50 countries, including Malaysia into three major clonal groups [29]. The 173 strains from five countries that were used for comparison in this study belonged to clonal groups A (n = 148) and C (n = 17) which were globally spread and clonal group B (n = 8) which was found in Europe, Africa and Asia [29] (Figure 3). One hundred and fifty one of these strains originated from 10 shigellosis outbreaks (O1 – O10) in Taiwan between 1996 and 2004. Another 22 strains were collected from nine epidemiologically unrelated episodes (E1 – E9) in Taiwan, China, Indonesia, Vietnam and Cambodia between 1998 to 2005 [16,18]. Based onVNTR loci SS1 and SS6, the Malaysian strains in the present study also belonged to these three major clonal groups. These observations suggest the *S. sonnei* strains circulating in Malaysia belonged to different clones that were spread worldwide. Although our observation may not represent the precise clonal structure of *S. sonnei* in Malaysia due to the limited sample size, the present study has nevertheless demonstrated the wide genetic diversity of *S. sonnei* circulating in this country.

## Conclusions

Shigellosis is endemic in Malaysia. Our results suggested that *S. sonnei* circulating in Malaysia were heterogeneous and generally derived from different clones. MLVA based on seven selected VNTR loci was rapid, reproducible and highly discriminative and therefore may complement PFGE for routine subtyping of *S. sonnei*.

## Competing interests

The authors declare that they have no competing interests.

## Authors’ contributions

XPK carried out the experiments, analysis and interpretation of data and drafted the manuscript. CSC provided the information for VNTR primers and participated in design and interpretation of data and helped in drafting of the manuscript. HW provided partial funding for the project and helped in editing of the manuscript. NA co-supervised the project, and participated in the design of project, and helped in drafting of the manuscript. Norazah A provided some of the strains and helped in editing the manuscript. KLT conceived study, participated in its design, coordinated and supervised the project and co-wrote the manuscript. All authors read and approved the final manuscript.

## Pre-publication history

The pre-publication history for this paper can be accessed here:

http://www.biomedcentral.com/1471-2334/12/122/prepub

## Supplementary Material

Additional file 1 **Information on the 173 S. sonnei strains from five other countries.** Information provided are extracted from previous study by Chiou et al. (2006), Liang et al. (2007) and Filliol-Toutain et al. (2011). Click here for file
