# Supplementary material for: Characterization of Shigella sonnei in Malaysia, an increasingly prevalent etiologic agent of local shigellosis cases
Source: BMC Infect Dis. 2012 May 20;12:122. doi: 10.1186/1471-2334-12-122 (PMC3420240; doi:10.1186/1471-2334-12-122)
Supplement: Additional file 1 — Information on the 173 S. sonnei strains from five other countries. Information provided are extracted from previous study by Chiou et al. (2006), Liang et al. (2007) and Filliol-Toutain et al. (2011). [file 1471-2334-12-122-S1.pdf]

Additional file 1: Information on the 173 *S. sonnei* strains from five other countries

Outbreaks (O)/ Unrelated episodes (E)[a,b]	Legend in Figure 3	Number of strain[a,b]	Year[a,b]	MLVA type[b]	Global clonal group[c]
O1	O1	7	1996	SS26.73	A
O2	O2	9	1998	SS26.21	A
O3	O3_1	6	1998	SS26.18	A
	O3_2	2	1998	SS26.74	A
O4	O4_1	5	1998	SS26.21	A
	O4_2	1	1998	SS26.75	A
O5	O5_1	3	2000	SS26.1	A
	O5_2	3	2000	SS26.129	A
	O5_3	43	2000	SS26.66	A
O6	O6_1	1	2001	SS26.102	C
	O6_2	16	2001	SS26.3	C
O7	O7_1	22	2001	SS26.1	A
	O7_2	2	2001	SS26.111	A
	O7_3	2	2001	SS26.125	A
	O7_4	1	2001	SS26.130	A
O8	O8	6	2001	SS26.1	A
O9	O9_1	7	2002	SS26.1	A
	O9_2	6	2002	SS26.125	A
	O9_3	1	2002	SS26.142	A
O10	O10	3	2003*	SS26.7	B
		5	2004*	SS26.7	B
E1	E1_1	3	1998	SS26.18	A
	E1_2	1	1998	SS26.74	A
E2	E2	1	1999	SS26.61	A
E3	E3	1	2004	SS26.58	A
E4	E4	7	2003	SS26.23	A
E5	E5	1	2004	SS26.54	A
E6	E6	1	2004	SS26.70	A
E7	E7	1	2004	SS26.69	A
E8	E8_1	3	2004	SS26.17	A
	E8_2	1	2004	SS26.68	A
E9	E9	2	2005	SS26.59	A

a: Chiou *et al.* (2006)

b: Liang *et al.* (2007)

c: Filliol-Toutain *et al.* (2011)

*: December, 2003 to January, 2004
